# Case Report: Complete heart block as a manifestation of cardiac metastasis of oral cancer

**DOI:** 10.12688/f1000research.26438.2

**Published:** 2020-11-11

**Authors:** Andrianto Andrianto, Eka Prasetya Budi Mulia, Denny Suwanto, Dita Aulia Rachmi, Mohammad Yogiarto

**Affiliations:** 1 Department of Cardiology and Vascular Medicine, Airlangga University - Dr. Soetomo General Hospital, Surabaya, East Java, 60286, Indonesia

**Keywords:** tongue cancer, cardiac metastasis, complete heart block, case report

## Abstract

Metastatic tumors of the heart presenting with complete heart block (CHB) is an extremely uncommon case. There are no available guidelines in managing CHB in terminal cancer. Permanent pacemaker implantation in such cases is a challenge in terms of clinical utility and palliative care.

We report a case of a 24-year-old man suffering from tongue cancer presenting with CHB. An intracardiac mass and moderate pericardial effusion were present, presumed as the metastatic tumor of tongue cancer. We implanted a temporary pacemaker for his symptomatic heart block and cardiogenic shock, and pericardiocentesis for his massive pericardial effusion. We decided that a permanent pacemaker would not be implanted based on the low survival rate and significant comorbidities.

Multiple studies report a variable number of cardiac metastasis incidence ranging from 2.3% to 18.3%. It is rare for such malignancies to present with CHB. The decision to implant a permanent pacemaker is highly specific based on the risks and benefits of each patient. It needs to be tailored to the patient’s functional status, comorbid diseases, prognosis, and response to conservative management.

## Introduction

Cardiac metastasis is the least common presentation in malignant cancer. Primary cardiac tumors are also rare (on postmortem analysis, commonly between 0.01% to 0.1%). However, the frequency of secondary metastatic tumors to the pericardium, myocardium, great vessels, and coronary arteries are between 0.7% to 3.5% in the general population and up to 9.1% in patients with a history of malignancies ^1^.

Complete heart block (CHB) as the primary clinical presentation of heart metastasis is very unusual ^2^. There are currently no guidelines for the management of CHB in terminal stage of cancer.

We report a case of CHB caused by cardiac metastasis and review the literature to further help the management of our patient.

## Case presentation

A 24-year old Asian man was admitted to the cardiology department with CHB and hypotension. The patient was a chef and had a history of tongue cancer for six months, and had undergone 30 cycles of radiotherapy. A week before, the patient came to the emergency department (ED) because of oral bleeding and general weakness. The patient denied any history of cardiovascular disease.

**Figure 1.**
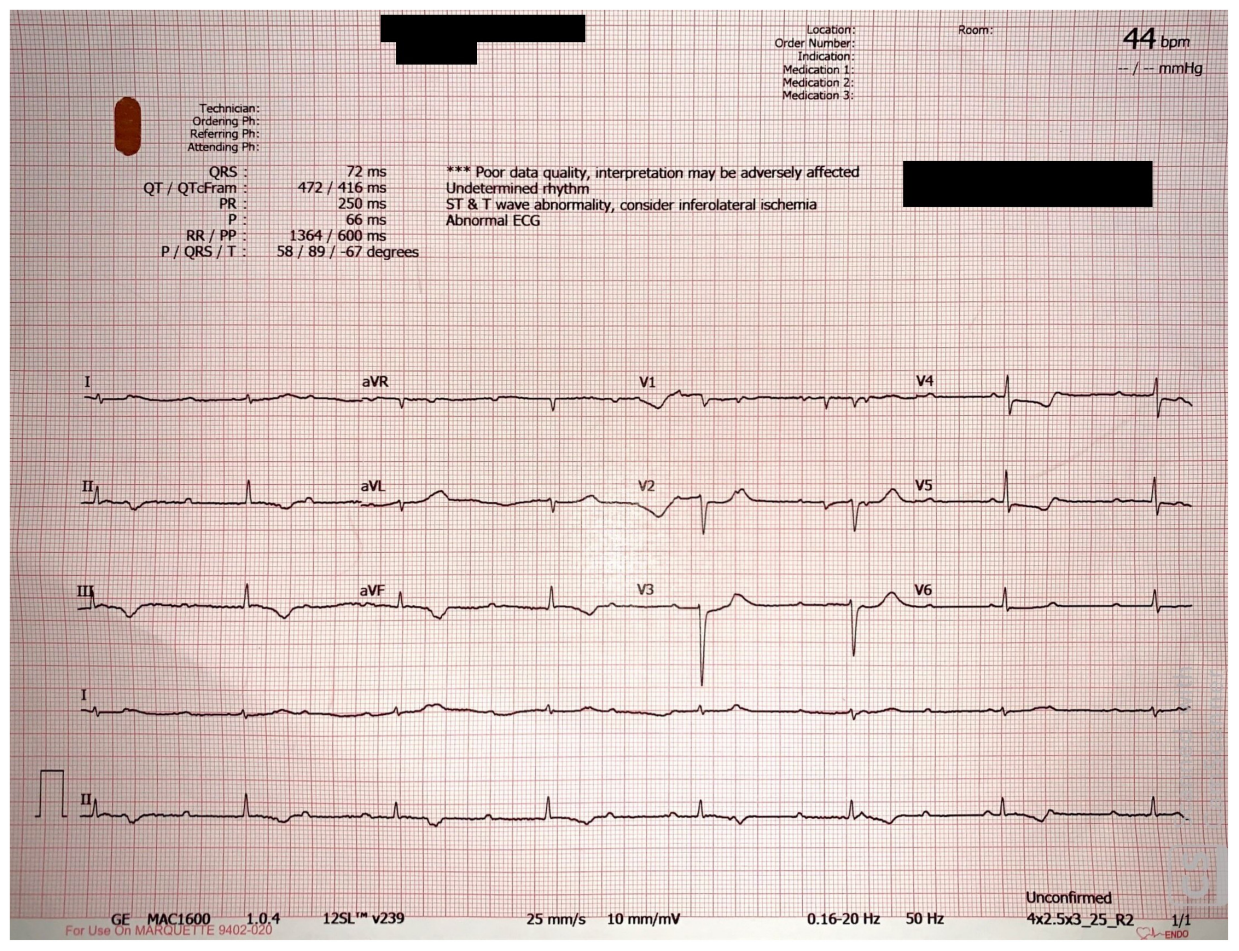
Electrocardiography on the first day of consultation showed complete heart block.

**Figure 2.**
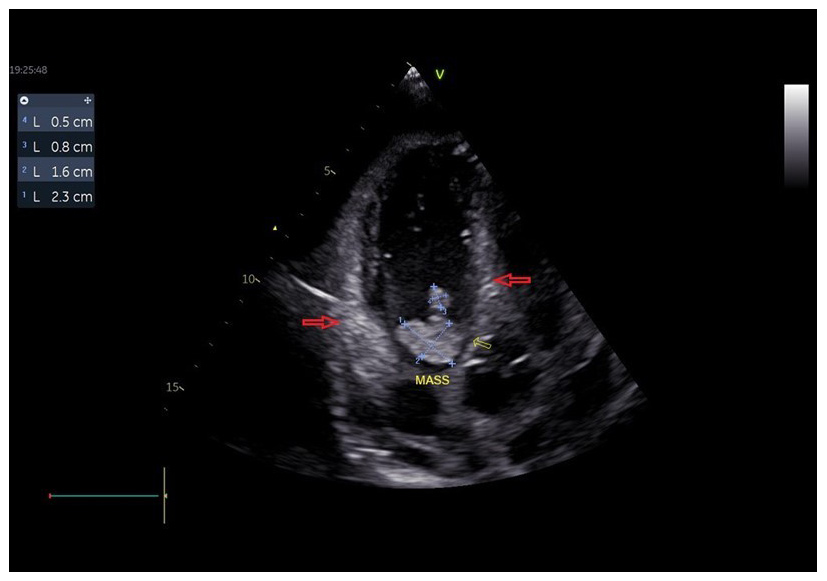
Echocardiography showed the presence of a mass in the right atrium and septal leaflet of tricuspid (yellow arrow). Hyperechoic areas were found in the annulus of tricuspid, lateral wall of right atrium and right ventricle, and interventricular septum (red arrows).

**Figure 3.**
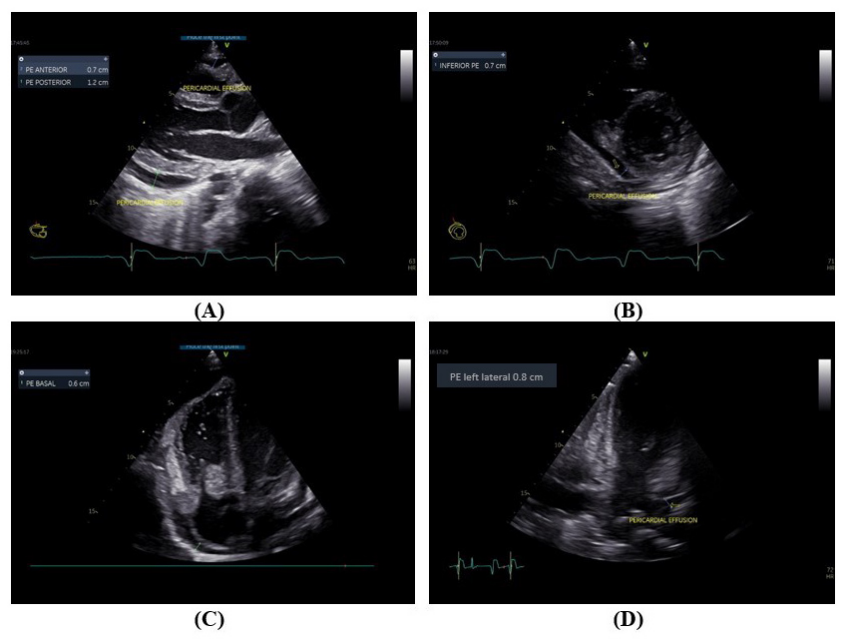
Pericardial effusion was found in ( **A**) anterior, posterior, ( **B**) inferior, ( **C**) base, and ( **D**) left-lateral of the heart.

The patient was presented with chest discomfort and general weakness. He was hypotensive and bradycardic with a blood pressure of 80/40 mmHg, regular heart rate of 44 beats per minute, respiratory rate of 18 breaths per minute, and oxygen saturation of 97% on room air. Chest auscultation was clear, and no murmurs heard. Electrocardiogram showed CHB with a junctional escape rhythm at 44 bpm ( Figure 1). Echocardiography showed normal left ventricle kinetic, normal left ventricular ejection fraction (62%), and normal right ventricle systolic function. There were moderate pericardial effusion and intracardiac masses (2.1 × 0.9 cm and 1.8 × 0.8 cm) in the right atrial and septal leaflet of tricuspid. Hyperechoic areas in the annulus of tricuspid, lateral wall of right atrium and right ventricle, and interventricular septum were also found in an echocardiogram ( Figure 2 and Figure 3). Laboratory finding revealed anemia (hemoglobin 8.5 g/dL; normal range 13.3-16.6 g/dL), leukocytosis (white blood count 18,470/mL; normal range 3,370-10,000/mL), hypoalbuminemia (albumin 2.6 g/dL; normal range 3.4-5.0 g/dL), hypokalemia (potassium 3.4 mmol/L; normal range 3.5-5.1 mmol/L), and hypercalcemia (calcium 16.2 mg/dL, corrected calcium 16.9 mg/dL; normal range 8.6-10.3 mg/dL).

Previous magnetic resonance imaging (MRI) of head and neck, six months before this admission, revealed malignant tongue mass (staging AJCC 2010 of lip and oral cavity mass: T4N1Mx) and bilateral nasal cavity thickening. Multi-slice computed tomography (CT) scan of the head revealed an enhancing mass 1×1.3×1.5 cm at the base of the tongue and multiple lymph node enlargements subcentimeter in the upper and lower paratracheal. Histopathology examination of tongue biopsy confirmed poorly differentiated squamous cell carcinoma.

A temporary pacemaker was immediately implanted as the patient showed symptomatic heart block and cardiogenic shock. After general supportive treatment, including intravenous dopamine administration, electrolyte imbalance correction, supportive treatment of general weakness condition, anemia, hypoalbuminemia, and infection, the patient showed improvement in general condition. No hemodynamic instability was observed when temporary pacemaker was turned off. However, the CHB persisted despite electrolyte imbalance correction.

Multidiscipline team discussions involving electrophysiologist, otolaryngologist, and internist, resulted in decision to focus more on palliative care for this patient. Considering the poor prognosis of this cancer, risk of permanent pacemaker (PPM) implantation, and severe comorbidities, we decided not to implant a PPM after acquiring the patient’s and his family’s consent.

Considering the risk of infection in prolong use of temporary pacemaker, on the 7 ^th^ day the temporary pacemaker was extracted. 

**Figure 4.**
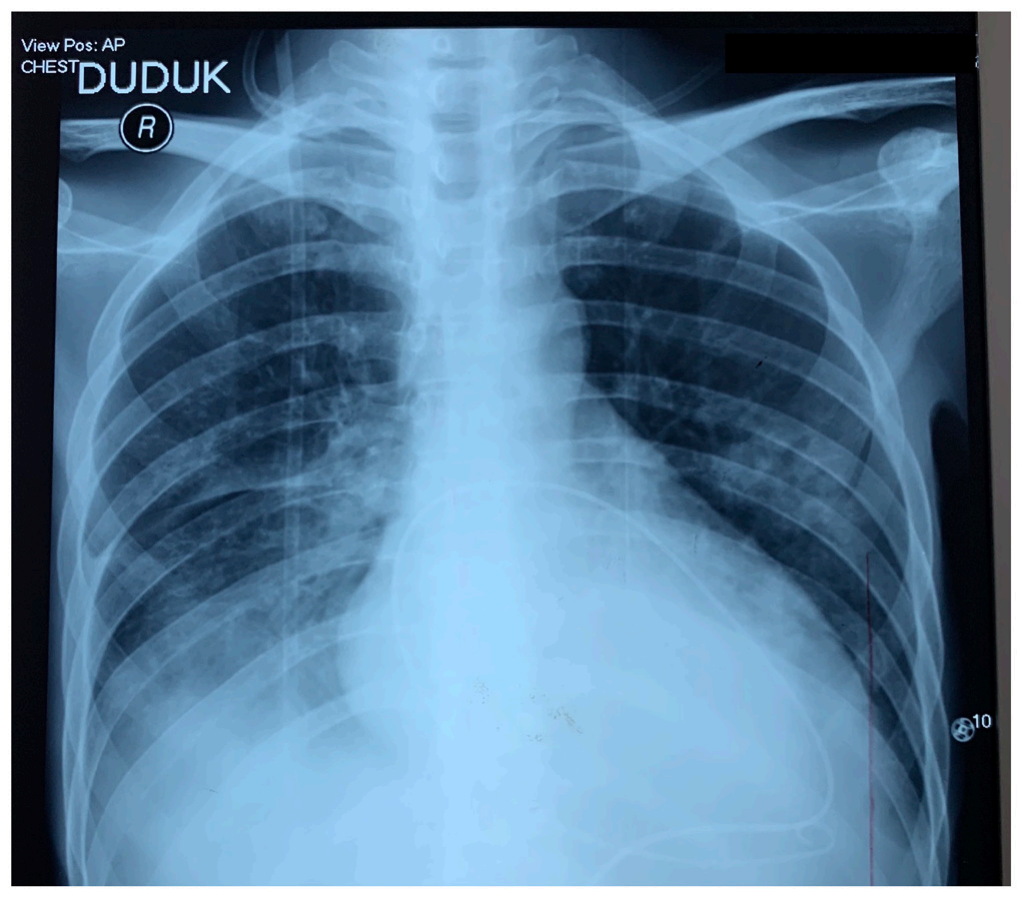
Chest X-ray on the 14 ^th^ day of treatment showed left parahilar ground glass appearance with suspicion of lung metastasis and pleural effusion. Pericardial fluid pigtail was already inserted for drainage of pericardial effusion.

On the 14 ^th^ day of admission, the patient developed pleural effusion, and worsening pericardial effusion with echocardiogram showed massive pericardial effusion and sign of tamponade. Further chest X-ray evaluation on the 14 ^th^ day showed left parahilar ground glass appearance with suspicions of lung metastasis and pleural effusion ( Figure 4). Pericardiocentesis was then performed with pericardial fluid showing hemorrhagic typical for malignant disease.

The patient died due to respiratory failure and septic shock 20 days after showing the first symptoms of cardiac metastasis.

## Discussion

From the literature, we acquired a total of 14 articles regarding heart block as a manifestation of cardiac metastasis, as summarized in Table 1 ^2– 15^. Oral cavity, uterus, and thyroid are the most common primary cancers that metastasize to the heart. Squamous cell carcinoma was the primary histologic finding. Heart metastasize may be present with clinically silent symptoms to an alarming presentation of hemoptysis and syncope. Locations of metastasis were mostly in the right ventricle, supporting the hypothesis of the hematologic spread of cancer cells. PPM implantation was performed in 10 cases, yet only one case reported a significant lifespan after PPM implantation.

**Table 1.** Literature review of case reports regarding heart block caused by cardiac metastasis.

Article	Age (years)	Primary site of cancer	Type of cancer cell	Location of cardiac metastasis	Sign / symptoms	ECG findings	Other cardiac manifestation	PPM implantation	Type of PPM	Reversibility of heart block	Treatment of cardiac metastasis	Diagnostic tools to detect cardiac metastasis	Lifespan after cardiac manifestation
Buckberg and Fowler, 1961 ^3^	42	bronchogenic carcinoma	adeno carcinoma	interventricular septum, anterior wall of left ventricle	shortness of breath, cough with blood	complete heart block	intracardiac mass	no	No	no	none	postmortem autopsy	6 weeks
Clifford *et al.*, 2003 ^8^	64	lymph	follicular small cell cleaved lymphoma	interventricular septum and RV anterior wall	nausea, diaphoresis, and dizziness	complete heart block with a ventricular escape	RV mass	yes	dual chamber	yes	chemotherapy	echocardiogram	2 years, still alive
Mocini *et al.*, 2005 ^9^	44	lung	malignant neoplasm	interventricular septum, left ventricular wall	syncope	complete heart block	mild pericardial effusion	yes	dual chamber	no	none	postmortem autopsy	3 days
Ferraz *et al.*, 2006 ^10^	63	uterus	squamous cell carcinoma	interventricular septum and RV	fatigue and dyspnea on mild exertion	complete heart block	pericardial effusion, RV mass	yes	permanent atrioventricular epimyocardial pacemaker	no	surgery for RV mass	echocardiogram, CT	4 months
Ozyuncu *et al.*, 2006 ^11^	56	right thigh	malignant melanoma	interventricular, interatrial septum	nausea, vomiting, presyncope	complete heart block	mitral regurgitation, pericardial effusion	yes	VDD	no	chemo- immunotherapy	CT thorax, echocardiogram	2 months, still alive
Knowles *et al.*, 2007 ^12^	42	right maxillary sinus	right maxillary sinus	not stated	syncope	complete heart block with a ventricular escape	pericardial effusion	no	no	yes	chemotherapy	echocardiogram, CT	1 year
Rathi *et al.*, 2008 ^13^	67	skin	malignant melanoma	all myocardial walls	asymptomatic	complete heart block, junctional escape	pericardial effusion	no	no	intermittent complete heart block	chemotherapy	echocardiogram, MRI	not stated
Lin *et al.*, 2015 ^14^	74	thyroid	papillary thyroid carcinoma	RVOT	exertional dyspnea and palpitations	first-degree atrioventricular block and subsequently a new intermittent complete atrioventricular block	RVOT mass	yes	not stated	no	none	CTA, C MRI, PET Scan	not stated (not long after discharge)
Yoneda *et al.*, 2016 ^15^	53	gingival	squamous cell carcinoma	atrial septum, left ventricle, AV node	cough, syncope	complete heart block, ventricular fibrillation	none	no	No	no	chemotherapy	postmortem autopsy	4 weeks
Park *et al.*, 2016 ^4^	54	right leg	leiomyosarcoma	interventricular septum	dizziness and dyspnea	complete heart block and idioventricular escaped rhythm of bifascicular block morphology	VT, intraventricular mass	yes	dual chamber	no	palliative chemotherapy	MRI	3 months
Yoshihiro *et al.*, 2017 ^5^	57	thyroid	squamous cell carcinoma	interventricular septum	cough and shortness of breath	complete heart block	RV mass	yes	not stated	no	chemoradiotherapy	FDG-PET	25 days
Kansai *et al.*, 2007 ^6^	56	lung	adenocarcinoma	interventricular septum	dull pain, presyncope	complete heart block	LV mass	yes	not stated	no	none	echocardiogram, CT, postmortem autopsy	19 days
Kumar *et al.*, 2018 ^2^	28	tongue	squamous cell carcinoma	interventricular septum	syncope	complete heart block	none	yes	not stated	no	none	PET Scan	5 days
Cho *et al.*, 2018 ^7^	70	oral cavity	squamous cell carcinoma	interventricular septum	dizziness	complete heart block with a ventricular escape	none	yes	DDD	no	palliative chemotherapy	FDG PET	not stated

The prevalence of cardiac metastasis, in general, is arguably low. However, multiple studies reported a variable number of cardiac metastasis incidence ranging from 2.3% to 18.3%. The prevalence of malignancy originating from the oral cavity was 5.3%. The involvement of pericardium made up two-thirds of all cardiac metastasis. Myocardium and endocardium involvement each made up one-third of all cardiac metastasis. Only 5% involved the endocardium. The most common site of metastasis for squamous cell carcinomas is epicardium (41.4%) ^16^.

Myocardial infiltration by cancer cells may present with arrhythmias, such as atrial flutter or fibrillation, premature beats, or ventricular arrhythmias. Conduction system involvement may induce a various degree of atrioventricular blocks ^16^.

The presence of right atrial mass in our patient supports the possibility of hematologic spreading of metastatic cancer cells into the endocardium. Pericardial effusion may represent metastasize or inflammatory reaction toward the malignancy. The presence of CHB suggests the infiltration of the heart conduction system.

Valves are an uncommon site for metastasis because of the absence of vessels in the physiological valvular stroma and the constant cusp motion. Bussani *et al*. reported, out of over a thousand of post-mortem examination, there was only one case of valve involvement ^16^. The mass in the septal leaflet of tricuspid valves appeared in echocardiography examination in this patient showed valve involvement of cardiac metastasis.

Asymptomatic in the early stages, cardiac metastasis could lead to a wide range of signs and symptoms, such as cardiac failure, conduction disturbances, angina, and pain as it progresses. Disruption of the cardiac conduction system by cardiac metastases can lead to lethal arrhythmias, including atrial fibrillation with a rapid ventricular response, CHB, or ventricular fibrillation ^1^. From the literature, we acquired 14 cases reporting CHB as a manifestation of cardiac metastasis originating from various malignancies, three of which are from the oral cavity.

Cardiac masses in our case presented with features favoring tumor, such as echo density similar to myocardium, normal wall motion, valvular lesion, no history suggestive of coronary artery disease, and a clinical history of oral cancer as primary site suspected to metastasize to the heart.

Cardiac MRI is the best imaging modality and, along with positron emission tomography scanning, are mostly used in investigating the extent of infiltration by malignant cells ^2, 17^. Patients with specific cardiac devices, such as pacemaker and defibrillators, would be disqualified from undergoing MRI, an important consideration given the frequency at which arrhythmia complicates cardiac metastasis ^17^. The temporary pacemaker implanted during the early stages of CHB made MRI impractical for our patient.

In our patient, CHB was initially thought to be the result of electrolyte imbalance. However, as the electrolyte was restored to its normal level without any improvement of CHB, it suggested that CHB was caused by infiltration of the metastatic cell to the conduction system of the heart. The presence of hemorrhagic pericardial effusion supports the suggestion of pericardial metastasis. Regardless of the lack of histological confirmation, we suggest that this case was cardiac metastasis diagnosed antemortem.

Predominantly, after a reversible or transient cause of bradycardia is excluded, cardiac pacing indication is decided by bradycardia severity instead of its etiology. The European Society of Cardiology (ESC) guidelines state that some types of persistent bradycardia require permanent pacing. In acquired AV block, pacing is indicated in patients with second-degree type 2 or third-degree AV block regardless of symptoms (class I) ^18^. Similar to the ESC guidelines, the American College of Cardiology/American Heart Association/Heart Rhythm Society guidelines also state that patients with transient or reversible causes of AV block should receive medical and supportive treatment if necessary, including temporary transvenous pacing, prior to confirmation of the need for permanent pacing (class of Recommendation/COR I). In addition, for patients with acquired CHB not associated with physiologic or reversible etiology, permanent pacing is recommended regardless of symptoms (COR I) ^19^.

In our case, we assume that the CHB was persistent after trying to correct possible external causes, such as hypercalcemia and hypokalemia, and after a sufficient waiting period. Moreover, it is still also unknown whether CHB would resolve after cancer treatment ^2, 20^. Our literature review showed that there are only two cases of metastatic CHB that are reversible after undergoing chemotherapy for cardiac metastasis ^8, 12^, while several other cases showed that CHB is irreversible ^2– 7, 9– 11, 13– 15^. The short life expectancy in patients with metastatic CHB also makes it difficult to follow up on CHB reversibility. Our literature review showed only one metastatic CHB case reported a significant lifespan more than two years after PPM implantation ^8^, while other cases reported a lifespan of no longer than one year ^2– 7, 9– 15^.

Our patient was expected to continue the radiotherapy cycle. Radiotherapy (RT) itself can induce pacemaker malfunction. Software impairments are the primary manifestation of malfunction during RT. This will perhaps lead to pacemaker reset, leaving only device basic function. Radiation dosage appears to contribute less to inducing pacemaker malfunctions than the beam energy of the RT ^21^.

For patients with indications for permanent pacing but accompanied with significant comorbidities or who are anticipated having a shortened lifespan because of terminal progressive illness, the implantation of a PPM should not be performed if it is unlikely to deliver significant clinical benefits or if it hinders the main therapy for the patient’s goal of care. Even though pacemaker implantation risks are rather low, the risk-benefit ratio is not favorable if the possible benefit is also quite low ^19^. Thus, after discussing with our patient and his family, we decided not to implant a PPM after acquiring the patient’s and his family’s consent.

## Conclusion

CHB in a patient with oral cancer should increase the physician's suspicion of cardiac metastasis. It is rare for such malignancies to present with CHB. Temporary pacemaker should be considered for patients presenting with CHB and unstable hemodynamic before deciding permanent pacemaker implantation. The decision to implant a permanent pacemaker is highly specific based on the risks and benefits of each patient. It needs to be tailored to the patient’s functional status, comorbid diseases, prognosis, and response to conservative management. Even though the pacemaker implantation risks are rather low, the risk-benefit ratio is not favorable if the possible benefit is also quite low. A permanent pacemaker was not implanted in our patient because of the poor prognosis, severe comorbidities, and low expected lifespan.

## Consent

Written informed consent for publication of their clinical details and/or clinical images was obtained from the patient’s parent.

## Data availability

All data underlying the results are available as part of the article and no additional source data are required.
