# Supplementary material for: Case Report: Complete heart block as a manifestation of cardiac metastasis of oral cancer
Source: F1000Res. 2020 Nov 11;9:1243. Originally published 2020 Oct 15. [Version 2] doi: 10.12688/f1000research.26438.2 (PMC7682501; doi:10.12688/f1000research.26438.2)
Supplement: Peer review history

# Peer review: Reviewer response for version 2

**DOI:** 10.5256/f1000research.30564.r74285

**Published:** 2020-11-19

**Authors:** Pieter A.F.M. Doevendans

**Affiliations:** 1 Department of Cardiology, Experimental Cardiology Laboratory, Division Heart and Lungs, University Medical Center Utrecht, Utrecht, The Netherlands

General 

 Interesting case report, there are only text and grammar issues. Well written, with supporting images. The case deals with a young adult treated for oral carcinoma who developed a symptomatic total AV block. The block persisted after restoration of the electrolyte dysbalance and is due to local tumour growth based on metatstasis. A conservative approach is chosen after balancing pros and cons of permanent pacemaker therapy. 

 In the discussion it would be nice to explain why no histology on pericardial fluid was performed. Maybe because of lack of any consequences for the patient or the treatment. 

 Textual corrections in detail for the authors:

 Masses in the right atrium and septal leaflet of the tricuspid valve.

 Showing hemorrhagic fluid

 Heart metastasis may be present without symptoms (silent) or show alarming symptoms .... 

 PE may represent pericarditis carcinomatosa or inflammatory reaction. 

 Post mortem examinations 

 metastatic cells

Are enough details provided of any physical examination and diagnostic tests, treatment given and outcomes?

Yes

Is the case presented with sufficient detail to be useful for other practitioners?

Yes

Is sufficient discussion included of the importance of the findings and their relevance to future understanding of disease processes, diagnosis or treatment?

Yes

Is the background of the case’s history and progression described in sufficient detail?

Yes

Reviewer Expertise:

Cardiology.

I confirm that I have read this submission and believe that I have an appropriate level of expertise to confirm that it is of an acceptable scientific standard.

# Peer review: Reviewer response for version 2

**DOI:** 10.5256/f1000research.30564.r74597

**Published:** 2020-11-19

**Authors:** Radityo Prakoso

**Affiliations:** 1 Department of Cardiology and Vascular Medicine, Faculty of Medicine, Universitas Indonesia, Jakarta, Indonesia

I think the author has revised all the suggestions and there is nothing more to be fixed from my point of view.

Are enough details provided of any physical examination and diagnostic tests, treatment given and outcomes?

Yes

Is the case presented with sufficient detail to be useful for other practitioners?

Partly

Is sufficient discussion included of the importance of the findings and their relevance to future understanding of disease processes, diagnosis or treatment?

Partly

Is the background of the case’s history and progression described in sufficient detail?

Yes

Reviewer Expertise:

Pediatric Cardiology and Congenital Heart Disease

I confirm that I have read this submission and believe that I have an appropriate level of expertise to confirm that it is of an acceptable scientific standard.

# Peer review: Reviewer response for version 1

**DOI:** 10.5256/f1000research.29188.r73101

**Published:** 2020-10-27

**Authors:** Radityo Prakoso

**Affiliations:** 1 Department of Cardiology and Vascular Medicine, Faculty of Medicine, Universitas Indonesia, Jakarta, Indonesia

Some typos and grammars should be evaluated. In the presentation, what does author mean about "improvement"? Why did TPM extracted by improvement but the complete heart block was actually not improving? I mean its 7th days were they sure that the worsening condition not due to the low cardiac output because of too low of heart rate? Maybe the author could add some suggestions of using medication to increase heart rate if there were significant reasons to "must take" the TPM?

 Conclusion

 So what does the author advise if one day there were similar patients came with complete heart block? Should the author suggest to implant the TPM or maybe using medication to increase the heart rate, such as sympathomimetic amines.

Are enough details provided of any physical examination and diagnostic tests, treatment given and outcomes?

Yes

Is the case presented with sufficient detail to be useful for other practitioners?

Partly

Is sufficient discussion included of the importance of the findings and their relevance to future understanding of disease processes, diagnosis or treatment?

Partly

Is the background of the case’s history and progression described in sufficient detail?

Yes

Reviewer Expertise:

Pediatric Cardiology and Congenital Heart Disease

I confirm that I have read this submission and believe that I have an appropriate level of expertise to confirm that it is of an acceptable scientific standard, however I have significant reservations, as outlined above.

# Response

**Published:** 2020-10-31

**Authors:** Andrianto Andrianto

**Affiliations:** Department of Cardiology and Vascular Medicine, Faculty of Medicine, University of Airlangga, Soetomo Academic and General Hospital, Indonesia

Some typos and grammars should be evaluated. In the presentation, what does author mean about "improvement"? Why did TPM extracted by improvement but the complete heart block was actually not improving? I mean its 7th days were they sure that the worsening condition not due to the low cardiac output because of too low of heart rate? Maybe the author could add some suggestions of using medication to increase heart rate if there were significant reasons to "must take" the TPM?

*Answer*: Thank you for your comment. We have revised come typos and grammar. We also added a clearer definition in general condition, supportive treatment including dopamine use, and reason the TPM was extracted in section *case presentation* paragraf 4-6.

 Conclusion

 So what does the author advise if one day there were similar patients came with complete heart block? Should the author suggest to implant the TPM or maybe using medication to increase the heart rate, such as sympathomimetic amines.

*Answer*: Thank you for your comment. We have added our suggestion of TPM use in such cases in section *conclusion* paragraf 1.
